# Insights into γ-irradiation effect on hole structure and conductivity of doped ethylene–propylene–diene rubber with different wheat husk fibers lengths

**DOI:** 10.1038/s41598-025-92154-x

**Published:** 2025-03-25

**Authors:** Hamdy F. M. Mohamed, Howayda G. Taha, Islam T. Zedan, Esam E. Abdel-Hady, Mohamed H. M. Hassanien, Hossam B. Alaa

**Affiliations:** 1 Physics Department, Faculty of Science, Minia University, P.O. Box 61519, Minia, Egypt; 2 Radiation Safety Department, Nuclear and Radiological Safety Research Center, Egyptian Atomic Energy Authority, Nasr City, Egypt; 3 Renewable Energy Science and Engineering Department, Faculty of Postgraduate Studies for Advanced Science, Beni-Suef University, P.O. Box 62511, Beni-Suef, Egypt; 4 Basic Science Department, Faculty of Engineering, Nahda University, P.O. Box 62513, New Beni Suef, Egypt; 5 El-Minia High Institute of Engineering and Technology, Minia, Egypt

**Keywords:** Ethylene–propylene–diene monomer rubber (EPDM), Wheat husk fibers (WHFs), Swelling, Positron annihilation lifetime (PAL), Free volume, AC conductivity, Materials science, Physics

## Abstract

In this paper, three samples of ethylene–propylene–diene monomer rubber (EPDM)/wheat husk fibers (WHF) with short (SW30), medium (EW30), and long (HW30) lengths of WHF were investigated as a function of γ-irradiation dose up to 300 kGy. The swelling coefficient Q of the toluene, benzene, and chloroform solvents was performed, and the Q reflects the ability of the polymer composites to absorb and retain a solvent within their structure. The conductivity of the examined composites has been computed, and their dielectric characteristics have been calculated. It was found that the dielectric constant exhibited frequency-dependent behavior indicating enhanced charge carrier mobility. The conduction mechanisms in the composites were found to be quantum mechanical tunneling and correlated barrier hopping, depending on the composite and irradiation dose. The γ-irradiation dose dependence of the free volume size, deduced from positron annihilation lifetime spectroscopy, is increasing for the SW30 composite while it decreases for both EW30 and HW30 composites. Increasing the free volume hole size is connected to the degradation effect on the SW30 samples. For EW30 and HW30, there is shrinkage of free volume in the composites due to crosslinking induced by γ-irradiation. Additionally, the nanoscopic properties derived from PAL spectroscopy are correlated with the macroscopic properties of the composites.

## Introduction

With the increasing need for clean and sustainable energy on a worldwide scale, it is more important than ever to ensure the safety and dependability of nuclear power. The complex interactions between several parameters, such as thermal stability, radiation resistance, and structural integrity, need novel materials to strengthen the nuclear safety basis. There is a rising global worry about the growing number of nuclear weapons and the potential abuse of radioactive materials; thus, innovative methods to strengthen safety measures are constantly being sought. Recently, there has been increased interest in using polymers in nuclear safety as a potential means of raising security requirements in several domains. Polymers provide unique properties that make them suitable for handling specific nuclear safety challenges due to their diverse chemical and physical properties. Using polymers in nuclear safety standards signifies a paradigm change, moving away from conventional materials and towards cutting-edge options with better endurance and performance. The effect of radiation on polymers can vary depending on the type of radiation (such as ionizing or ultraviolet) and the specific polymer composition. There are some general effects of radiation, such as ionization radiation (gamma rays or high-energy electrons), on polymers: (1) Chain scission, which involves the breaking of polymer chains, resulting in a decrease in molecular weight and a loss of mechanical strength, flexibility, and other physical properties of the polymer^1–3^.

The irradiation of polymers stands as a very useful manufacturing procedure as a substitute for more traditional chemical processes, which adjust several materials’ properties. Some polymers may undergo color changes upon radiation exposure^4–6^. This is particularly evident with ultraviolet (UV) radiation, which can induce photochemical reactions in polymers, resulting in discoloration or yellowing. In addition, radiation can affect the electrical properties of polymers. For instance, it can alter polymers’ dielectric constant, resistivity, and other electrical characteristics. This effect is crucial in applications involving radiation-sensitive electronic components or insulating materials^7–9^. Not all polymers are equally susceptible to radiation damage. Some polymers, such as certain fluoropolymers or polyimides, exhibit excellent radiation resistance due to their chemical structure and inherent stability. These polymers are often used in radiation-intensive applications such as nuclear power plants or nuclear safety^10,11^.

The properties of composite media in the nuclear field and in laboratory conditions can be studied with the use of the γ-ray transmission method. The method’s correct calibration is the issue at hand. Since there is currently no method that can match the accuracy of the γ-ray transmission method, an experimental approach is being carried out.

Innovative spectroscopic methods that provide previously unattainable insights into atomic structures and dynamics have been welcomed by researchers in their quest to comprehend the underlying characteristics and behaviors of materials at the nanoscale. Positron annihilation lifetime spectroscopy (PALS) is an effective technique that has grown in popularity in materials research. This accurate and safe method offers a unique view into the dynamics of the electron–positron interaction within various materials, holding the key to understand the complexities of molecular settings. Positron and electron are bonded together in polymers and form positronium (Ps) atoms at the final step of positron thermalization. The fact; that Ps is particularly trapped (restricted) in atomic scale open volume; explains why positron annihilation lifetime (PAL) is effective when exploring free volume or open hole qualities. The need for materials with customized characteristics is growing, and PALS approaches are becoming more and more critical for characterizing and optimizing material structures^12–14^.

It can be found that the free volume hole size correlates with several polymer physical characteristics^15,16^. On the other hand, optimizing the performance of polymers for specific purposes requires a thorough understanding of how they behave when exposed to solvents. The process by which a polymer matrix absorbs solvent molecules is known as swelling, which is essential to the characteristics and performance of the material^17^. A vital indicator of the degree of swelling is the swelling ratio, which is the change in the polymer’s volume or weight because of solvent absorption. Because swelling in polymers has ramifications for material science, chemical engineering, and biomaterials, it has attracted a lot of research. Examining the relationship between these variables and the swelling behavior that results, offer important insights into the interactions between polymers and solvents, and directs the logical design of new materials with specific swelling behaviors.

Due to their use in radiation shields and other radiation protection applications, studies on the electrical characteristics of polymer composites have garnered a lot of attention. Polymer ionic conduction is currently used to understand the nature of the charge transfer that is present in these substances. Dopants can be added to polymers to suitably alter their electrical characteristics^18^. Using various inorganic fillers may significantly enhance the mechanical, thermal, optical, electrical, and other properties of polymers^19^. These additives are widely used in the plastics industry to achieve the same levels of performance at a lower cost than would otherwise require considerably more expensive engineering plastics; their surface allows for easy surface bonding when a polymer is applied to it^20^.

Polymer composites have been created by combining several types of synthetic reinforcing filler with polymer to enhance their mechanical properties and achieve the desired features for real-world applications. The combination of natural fibers such as wheat husk fiber (WHF) and synthetic polymers such as ethylene–propylene–diene monomer rubber (EPDM) offers not only a great opportunity to utilize agro-based natural resources but also demonstrates the desired performance qualities from a more sustainable material that would otherwise pollute the environment if disposed of or burned. A good compounding procedure and efficient component mixing will result in better filler dispersion. Also, the length or the concentration of the filler can affect the properties of the composites^21^. Numerous efforts have been undertaken to enhance the filler-matrix interface bonding. WHFs are agricultural waste with the potential to be substantial, reasonably priced, and ecologically fillers derived from a renewable source. On the other hand, EPDM has high tensile strength, toughness, and age resistance, making it ideal for usage in various applications.

To better understand polymer applications in the field of nuclear safety, this research article examines their special qualities and possibilities for reducing dangers related to nuclear energy. Mohamed et al.^22,23^ investigated the free volume of EPDM/WHFs using the PAL spectroscopy. They studied the impact of the reinforced WHF concentrations in the range of 0–50 phr on the free volume structure of EPDM/WHF composites, along with the effect of γ-irradiation doses from 0 to 300 kGy. To continue our previous study on EPDM/WHFs composites, the aim of this work is to characterize the nanostructure of the free volume size, electrical properties, solvent swelling, and γ-ray transmission of EPDM/WHF composites with different lengths of WHF as a function of γ-irradiated doses. The free volume nanostructure of the present samples was studied using PAL spectroscopy. The electrical properties have been investigated by measuring the AC electrical conductivity and the dielectric properties as functions of frequency and irradiation doses. The effect of different solvents such as toluene, benzene, and chloroform was studied. The γ-attenuation coefficients were investigated using γ-ray transmission measurements. A correlation between the positron annihilation parameter and other results such as the ionic conductivity was investigated.

## Experimental and data analysis

Table 1 lists the chemical composites of the present samples. Ethylene–propylene–diene monomer (EPDM) rubber was provided from Uniroyal Chemical Co., Inc. of Naugatuck, USA. Stearic acid, Zinc Oxide, and Sulphur are supplied by Transport and Engineering Company, TRANCO, Alexandria, Egypt. These ingredients are of commercial grade and don’t require any additional purification. Paraffin wax was utilized. Toluene, benzene, and chloroform were the organic solvents employed for the swelling test and were purchased from Sigma-Aldrich, Egypt. Wheat husks were harvested from Upper Egypt’s; Beni-Suef region. To eliminate any remaining unwanted dirt, clay, or dust, the wheat husks were thoroughly cleaned many times with tap water before being dried for an entire night at 102 ± 2 °C. After being dried and processed mechanically in a rotary cutting mill, the wheat husks were automatically sieved into length-based categories. The fiber lengths that were examined can be categorized into three items: small SW (< 125 µm), medium EW (125–250 µm), and high HW (> 250 µm) as shown also in Table 1. The composite materials were prepared in a two-roll laboratory mill that was maintained at almost 50 °C. For every combination, the mill/roll speed ratio, nip gap, and number of passes remained constant. At a 1.25 mm mill opening, the samples were ground for a long enough time to distribute the fibers throughout the matrix. After mixing, the WHF was added, making sure to consider the compound’s flow direction so that most of the fibers were pointing in the same direction.

**Table 1:** The chemical composition blend ratios of ethylene propylene diene monomer/wheat husk fibers (EPDM/WHF).

Ingredients (phr)	SW30	EW30	HW30
EPDM	100	100	100
Zinc Oxide	5	5	5
Stearic acid	1	1	1
HAF^a^ (N-330)	30	30	30
Paraffin	5	5	5
WHF^b^	30	30	30
MBTS^c^	1	1	1
PβN^d^	1	1	1
Resin	5	5	5
Sulfur	2	2	2
Length of WHF, μm	< 125	125–250	250–500

^a^High abrasion furnace black.

^b^Wheat husk fibers.

^c^Dibenthiazyl disulfide.

^d^Phenyl-β-naphthylamine.

**Fig. 1:**
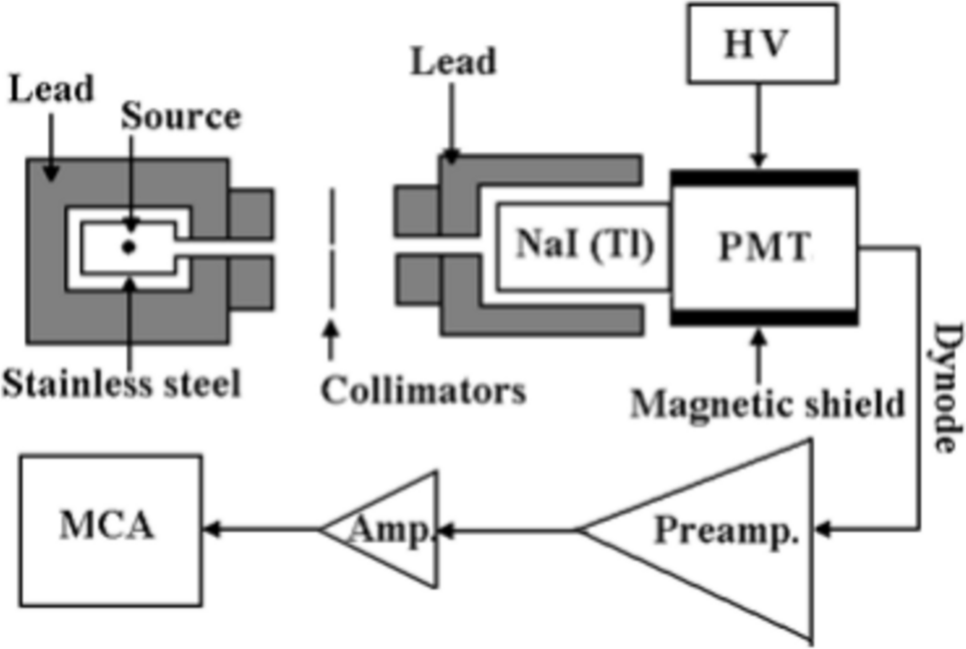
Schematic diagram of the experimental set-up for γ-ray transmission.

A gamma source was used to irradiate each sample. A ^60^Co source for γ-radiation was housed at the National Center for Radiation Research and Technology (NCRRT), Egyptian Atomic Energy Authority, Cairo, Egypt. The three studied Composites were irradiated at 10, 30, 50, 70, 100, 150, 200 and 300 kGy. A cylindrical 2.5 cm radius by 5 cm thick NaI(Tℓ) scintillation detector is part of the experimental setup used to study γ-ray transmission. A photomultiplier tube (PMT) model RCA 8850 is visually linked to the NaI(Tℓ) crystal. The photomultiplier and the crystal are encircled by a cylindrical Mμ-magnetic shield. Through an ORTEC (575A) amplifier, the dynode pulses are routed to an ORTEC multichannel analyzer (MCA) configured to 4096 channels. The detector is protected by a thin layer of metal that is secured by a lead stronghold that is 5 cm thick. Figure 1 shows a block diagram of the setup that was used in this investigation. A ^241^Am radioactive source having energy of 59.5 keV is placed in an appropriate lead container to produce a pin beam that has a diameter of 1 mm for γ-ray transmission. The NaI(Tℓ) scintillation detector, the source, and the sample itself are all placed on a stand so that their points of focus are on the identical direction. The MCA/PC spectroscopy framework is linked to the detector to collect and analyze the transmission data. The correlation table on which the tests are conducted has a shielded NaI(Tℓ) scintillation detector on one side and a protected γ-ray source shield-collimator on the opposite side. In between, the necessary source-detector distance is used to modify the location of an appropriate material holder.

The Lambert–Beer law states that a parallel beam of mono-energetic γ-rays is attenuated in the matter as^24^:

1 $${\text{I}} = {\text{I}}_{{\text{o}}} \exp \left( {\upmu _{{\text{L}}}\, {\text{x}}} \right).$$

where µ_L_ is the linear attenuation coefficient, x refers to the sample’s thickness, and I_o_ and I are both the incoming and outgoing γ-ray flux. The half value layer [x_1/2_ (HVL); or half thickness] is related to the linear attenuation coefficient, and it is the thickness of a certain material required to reduce the intensity of a γ-ray beam to half of its initial value^25^:

2 $${\text{x}}_{1/2} \left( {{\text{HVL}}} \right) = 0.693/\upmu _{{\text{L}}} ,$$

The mean free path (MFP), which is the average distance that a photon moves between interactions, is

3 $${\text{MFP}} = 1/\upmu_{{\text{L}}} .$$

Equations (1–3) are used to consider the physical characteristics of the three unirradiated samples, such as μ_L_, HVL, and MFP. In all counting measurements, the peak area determinations and the error resulting from counting statistics were maintained at less than 1%.

The positron source was ready by dropping about 20 µCi of aqueous ^22^NaCl on a thin Kapton® foil (7 µm thick). Following the drying of ^22^NaCl spots, it was sealed with a second piece of the same foil joined together with epoxy glue and left outside for a full day. About 10% of positrons were adsorbed by the Kapton® foil, mostly contributing to the short lifetime components^26,27^. Positron annihilation lifetime (PAL) was measured using a standard Fast–Fast coincidence setup. Since Kapton® is the polymer without positronium formation (long-lived component), it can be employed for determining the time resolution of the system^28^. The time resolution of the current PAL system is 240 ps. Standard specimens of synthetic fused silica (NMIJ CRM 5601-a) and polycarbonate (NMIJ CRM 5602-a) were supplied by the National Metrology Institute of Japan, National Institute of Advanced Industrial Science and Technology (AIST), for the purpose of calibrating the current PAL system^29,30^. For polymers and insulators with a Ps component that has a lifetime longer than 1 ns, the standard samples were meant to be used in the PAL technique to validate the measurement circumstances and regulate the precision of the measured data. This allowed the method to confirm the measurement conditions and control the precision of the collected data. The average lifetimes ${\tau }_{i}$ (i = 1 − 3) and their intensities *I*_*i*_, and the background of the spectrum *B*, have all been determined via non-linear least squares fits utilizing the PALSfit (Version 3.251) software^31,32^. Each positron lifetime spectrum with a total count of 3.0 × 10^6^ counts was analyzed into three lifetime components and with a positron source correction. The conventional discrete term analysis has been conducted using the standard LT10 (Version 10) software^33–35^. A relationship has been estimated by Tao et al.^36^ and Eldrup et al.^37^ between the observed o-Ps lifetimes τ_3_ and the radius of the free volume size *R* as:

4 $${\tau }_{3}=0.5{\left\{1-\frac{R}{{R}_{o}}+\frac{1}{2\pi }sin\left\langle \frac{2\pi R}{{R}_{o}}\right\rangle \right\}}^{-1},$$

where the thickness of the homogeneous electron layer when the positron annihilates is given by *ΔR* = 0.1656 nm and *R*_*o*_ = *R* + *ΔR*^38^. The free volume hole size *V* in nm^3^ is given as;

5 $$V = 4\pi R^{3} /3.$$

The swelling coefficient Q for different solvents (toluene, benzene, and chloroform) as solvent uptake was calculated as:

6 $$Q = \frac{\left({W}_{s} -{W}_{d}\right)}{{W}_{d}} \times100.$$

where *W*_*d*_ is referred to the weight of the dry polymer sample and *W*_*s*_ is the weight of the polymer sample after swelling in the solvent. The Q was calculated for the three samples (SW30, EW30, and HW30) irradiated with different irradiation doses 0–300 kGy. The samples were evacuated for more than 24 h to get the dried samples and then weight for getting their weight *W*_*d*_. Then the dried samples were immersed into each solvent and weighted to get the swelling weight *W*_*S*_ as a function of immersed time from 0 to 1440 min at 25 °C.

At room temperature (25 °C), the conductance *G* of each present sample was measured using a Hioki 3532 LCR meter in the frequency range from 50 Hz to 5 MHz. With the accuracy within ± 2%, the ionic conductivity σ was calculated as^39,40^;

7 $${\text{Ionic conductivity}}\, \upsigma ({\text{S}}/{\text{cm}}) = \frac{Gd}{A}$$

where *G* = 1/*R*_b_ (*R*_b_, is the bulk resistance), *d* is referring to the thickness of the sample and *A* is the cross-sectional area of the sample.

## Results and discussions

Ionizing radiation causes tiny molecular fragments to develop, chain scission and/or crosslinking, and changes to the free volume structure of polymers. Free volume changes, resulting from crosslinking and/or chain scission, can markedly influence the material properties^41,42^. In specific irradiation polymers, a rivalry between scission and crosslinking processes has been seen^43^. Both processes can occur at the same time, and which one takes precedence relies on the kind of polymer, the ambient conditions at the time of irradiation, and the total dose. The analysis of the PAL spectra using the PALSfit and LT10 programs^31,33^ deduce lifetime’s τ_1_, τ_2_, and τ_3_ with their intensities *I*_1_, *I*_2_, and *I*_3_, respectively. For all the samples, the shortest-lived component (τ_1_ ~ 0.125 ns and *I*_1_ = 19–26%) contained the contribution of spin antiparallel *para*-positronium (*p*-Ps); the intermediate one (τ_2_ ~ 0.300–0.389 ns and *I*_2_ = 61–88%) was due to the annihilation of positrons that did not form Ps; and the component with the lifetime τ_3_ = 1.93–2.61 ns and *I*_3_ = 10.18–17.36%) was attributed to the *o*-Ps pick-off annihilation^44,45^.

**Fig. 2:**
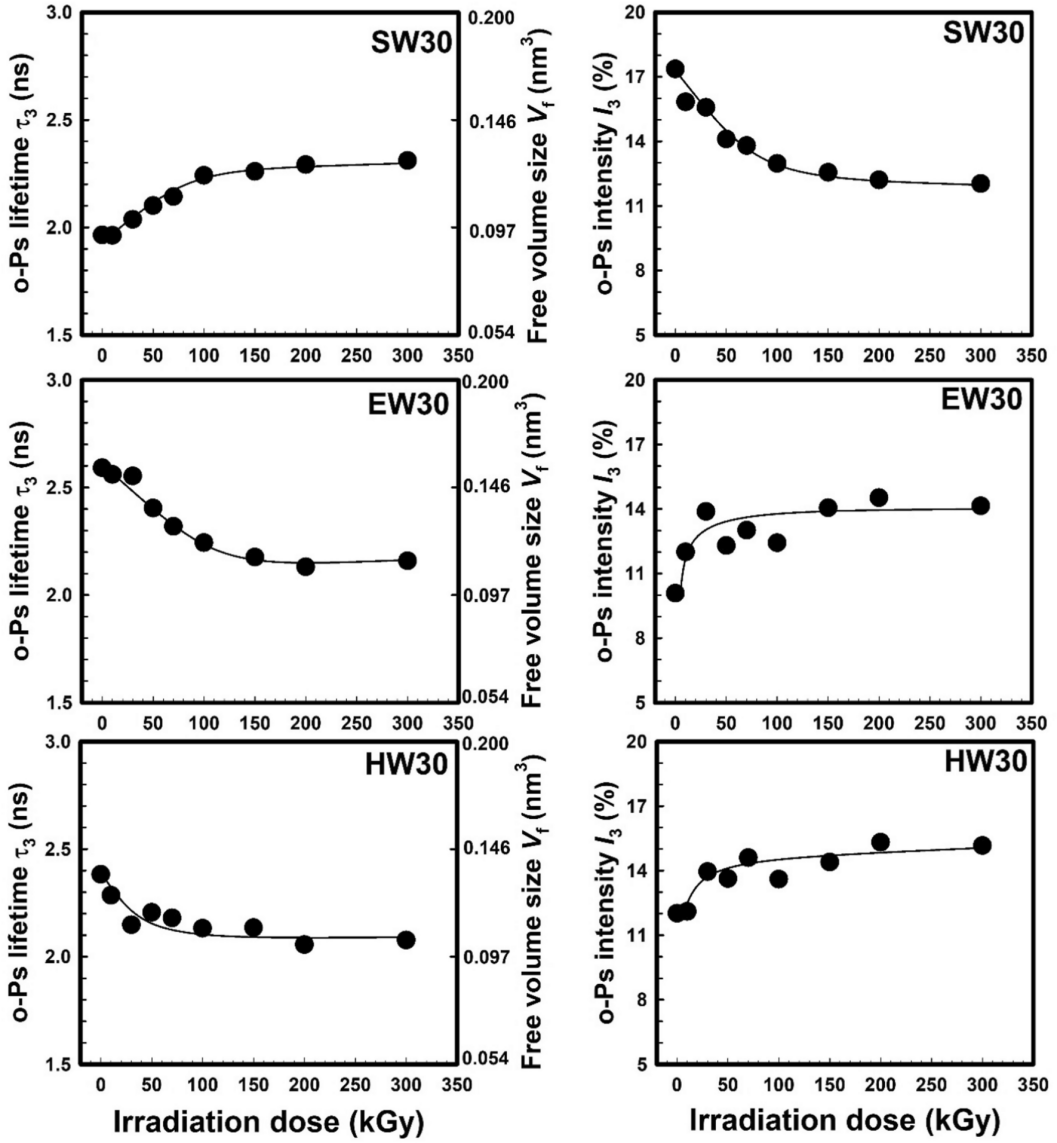
Lifetime τ_3_ and intensity *I*_3_ of *o*-Ps as a function of the irradiated dose for the SW30, EW30, and HW30 samples. The scale of the right ordinate in the left panel is the *o*-Ps hole volume size *V* deduced from Eqs. (4 and 5). The statistical error of each point is smaller than the plotting symbol. The solid line is drawn just for clarification.

Figure 2 shows the *o*-Ps lifetime τ_3_ and its intensity *I*_3_ as a function of the γ-irradiation dose for the three samples (SW30, EW30, and HW30). The right-hand ordinate of the left figures represents the spin parallel *ortho*-positronium (*o*-Ps) hole volume size *V* deduced from Eqs. (4 and 5). As clear from Fig. 2, the *o*-Ps lifetime τ_3_ (*o*-Ps free volume size *V*) increases with increasing the γ-irradiation dose up to 100 kGy and then it is leveled off for the SW30 samples with a shorter length of WHF. Increasing the free volume hole size at low irradiation dose can be connected to the degradation effect on the SW30 samples, while at high irradiation dose, the degradation effect leveled off and the free volume is unchanged. On the other hand, the *o*-Ps intensity *I*_3_ for the SW30 sample decreases exponentially with increasing the γ-irradiation dose. Recombination of positrons with one of the extra electrons in polymers can produce positronium. Positronium formation can be inhibited by free radicals generated by γ-irradiation because they can scavenge electrons produced by positrons^45^. Increasing the degree of crystallinity on the polymer can decrease the formation of positronium (lower *I*_3_) because of decreasing the amorphous part^46^. Also, the shorter chains resulting from chain scission by γ-irradiation also contribute to higher density (high crystallinity). So, decreasing the *o*-Ps intensity on the SW30 samples with γ-irradiation dose can be connected to both effects: formation of free radicals or an increase in the degree of crystallinity by γ-radiation. The major chain scission occurs when SW30 samples are exposed to radiation at 25 °C, and the short chains created in the amorphous stage align to form crystallites. The morphology of the newly formed crystalline part should be rather disordered\break containing large vacancies that can accommodate Ps. The current result is consistent with the findings of Ito et al.^41^, who discovered that larger vacancies are found by *o*-Ps in irradiated polytetrafluoroethylenes (PTFE) that are either cross-linked or degraded. Additionally, they found that when PTFE is exposed to radiation at 25 °C, main chain scission occurs, and the short chains generated in the amorphous region align to form crystallites. Additionally, removing a hydrogen atom from a nearby carbon atom may convert the peroxy radical produced at the polymer chain into a hydroperoxy function. Because the freshly created radical can react with an oxygen molecule on its part, this might start a chain reaction^47^.

For the polymer composite with medium length of WHF (EW30) and with long length of WHF (HW30) samples, the trends of *o*-Ps lifetimes τ_3_ (free volume hole *V*) and their intensities *I*_3_ are opposite compared with those of SW30 sample as also shown in Fig. 2. The *o*-Ps lifetime exhibits an exponential reduction as the γ-irradiation dosage increases, indicating shrinkage of the free volume due to crosslinking of the polymer chains by γ-irradiation. On the other hand, the *o*-Ps intensity *I*_3_ increases exponentially with irradiation dose because of increasing the free volume contents (amorphous region) in the EW30 and HW30. In addition, the irradiation process might induce the formation of voids or defects within the material^48^. These voids or defects can create additional free volume content in the material, leading to an increase in the *o*-Ps intensity *I*_3_. However, excessive void formation can be detrimental to the mechanical properties and overall performance of the material. The present data agrees with the data of Yang et al.^49^ who studied the γ-irradiation on low density polyethylene and polyvinyl alcohol. The free volume was influenced by the interplay between crosslinking density and the degree of crystallinity of the samples due to γ-irradiation. Increasing the crosslinking density resulted in a comparable decrease in crystallinity, which caused the molecular chains to become misaligned and promoted increased chain mobility (increasing *I*_3_). This, in turn, translated to an enlargement of the free volume within the polymer matrix. Nevertheless, the simultaneous increase in polymer crosslinking connections resulted in the formation of a denser network structure, which subsequently reduced the free volume (lowering τ_3_). So, crosslinking in the samples reduces the mobility of polymer chains, limiting their ability to move and resulting in a decrease in free volume. Also, the increased crosslinking density leads to a decrease in free volume due to the reduced amount of available space within the material.

The presence of natural fibers, such as WHF, chitosan, and other organic components, can influence the radiation-induced effects due to their interaction with the polymer matrix^50^. The fibers may act as physical barriers, affecting the penetration and distribution of radiation within the composite material. As seen from Fig. 2, the length of the WHF affects the nanostructure of the polymer composites that can be seen from the different PAL parameters (τ_3_, *I*_3_) for all the three samples. The free volume hole sizes for unirradiated samples of SW30, EW30, and HW30 are 0.092, 0.157, and 0.136 nm^3^, respectively. On the other hand, the positronium formation or the free volume contents for the unirradiated samples of SW30, EW30, and HW30 are 17.363, 10.082, and 12.013%, respectively. The effect of inhibition of *I*_3_ for the irradiated samples at 300 kGy was neglected, and the fractional of the free volume (~ *I*_3_. *V*) was calculated. It is found that the fraction of the free volume is in the sequence of EW30 < SW30 < HW30 indicating that the medium length of the WHF leads to a smaller fraction of the free volume in the polymer composite compared with those in the short and long lengths of the WHF loaded into EPDM. This result can be connected to the specific volume of the samples, where a smaller fraction of the free volume leads to high specific volume.

There are some additional details regarding the effects of the γ-irradiation dose on the free volume in the samples. This process leads to the creation of a three-dimensional network of interconnected polymer chains. The crosslinked network restricts the movement of polymer chains, reducing their ability to slide past one another. This decrease in chain mobility results in a decrease in free volume, as the available space for chain movement and reorientation is constrained. The crosslinked structure imparts greater stiffness and rigidity to the material. The formation of free radicals during irradiation can cause the cleavage of polymer bonds, leading to the formation of shorter polymer chains. The presence of shorter chains affects the overall polymer structure and can contribute to a decrease in free volume. Shorter chains have less freedom to move and occupy space within the material, resulting in a reduction in the available free volume (increase in density and material shrinkage). The combined effects of crosslinking and chain scission, along with other radiation-induced changes in the polymer matrix, can lead to an increase in material density. Crosslinking creates a denser network of polymer chains, while chain scission reduces the average molecular weight of the polymer^51^. The increase in density causes the material to shrink, as there is a reduction in the volume occupied by the polymer matrix. This shrinkage further contributes to a decrease in free volume. Fiber-polymer interactions: The presence of natural fibers, such as wheat husk fiber (WHF), in the composite material can influence the radiation-induced effects on free volume. The fibers can act as physical barriers that affect the penetration and distribution of radiation within the composite.

**Fig. 3:**
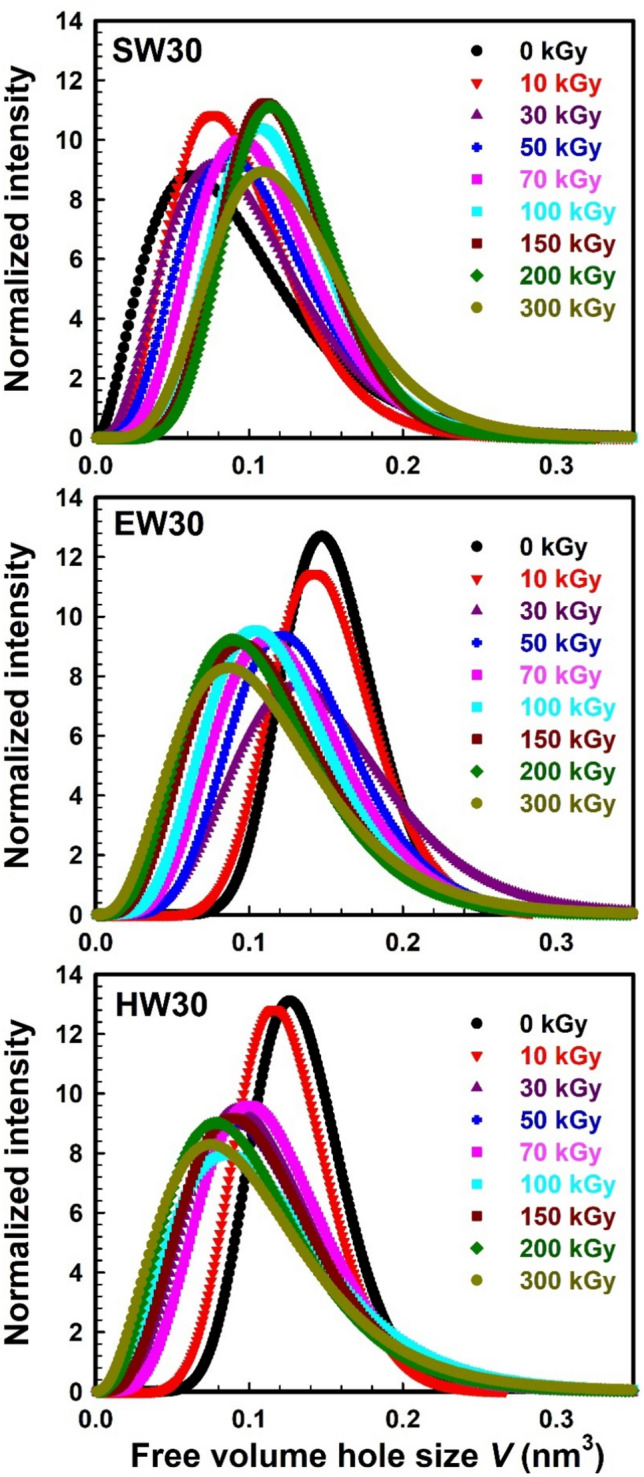
The free volume distribution as a function of the free volume hole size for the SW30, EW30, and HW30 samples at different γ-irradiated doses.

One of the main properties of the materials is the free volume distribution, which affects mostly the properties of the materials. Free volume distribution is responsible for the crystal structure of the samples and also for thermal and mechanical stability. So, it is interesting to study the free volume distribution in the samples, which can be obtained using LT10 program. Figure 3 shows the free volume distribution at different γ-irradiation doses for the SW30, EW30, and HW30 samples. It is clear from the SW30 figure that the free volume distribution shifted to a higher free volume size with increasing the γ-irradiation doses. This shift of the free volume distribution with γ-irradiation dose connected to the degradation effect occurred in the SW30 samples, which led to increase the free volume size. The width of the free volume distribution also gives interesting information. The free volume distributions became narrower (less broad) with increasing the γ-irradiation dose. It signifies that, during the irradiation effect, small free volumes come together to become larger free volume sizes, as it is accompanied by a decrease in the total number of smaller free volume sizes. It is indicative of the homogenization of the materials as the degradation proceeds. In addition, the decrease in *o*-Ps intensity might suggest the formation of electronegative groups. During the irradiation process, some electronegative groups, including polar groups, might graft with the sample’s molecular chains. These polar groups could inhibit the formation of positrons, which would lead to a reduction in the intensity of the *o*-Ps^52^. For the EW30 and HW30 samples (Fig. 3), by increasing the γ-irradiation dose, the free volume distributions shift to a smaller free volume size, which is related to the crosslinking of the polymer chains induced by γ-irradiation. The free volume holes shrink, which are interpreted in terms of crosslinking and aggregation of ions in sample microstructure. By increasing the γ-irradiation dose, chain scissions become predominant, and free volume of smaller sizes are created. On the other hand, the free volume distributions obtained for the irradiated samples after the 10 kGy irradiation dose are significantly broader than those for the un-irradiated and 10 kGy irradiation doses for both EW30 and HW30 samples. Such a trend suggests that the impact of radiation reinforcement on the polymer matrix’s free volume microstructure decreases following a high dose of radiation, which might be the result of damage to the interface and a lack of adhesion between the polymer matrix and WHF reinforcement. These γ-irradiation doses can create additional free volume content in the material, leading to an increase in the number of free volume holes.

Table 2 listed the obtained values of some γ-ray transmission properties as μ_L_, HVL, and MFP for the SW30, EW30, and HW30 composites using ^241^Am radioactive source (at 59.5 keV). It is clear from the table that the trends of μ_L_, HVL, and MFP are almost similar. The μ_L_ value for the EW30 is higher compared with those for the SW30 and HW30. With increasing the length (> 250 µm) or decreasing the length (< 125 µm) of the WHF leads to an increase in the µ_L_ value that decreases the HVL and MFP values. These increases in the µ_L_ and decreases in both HVL and MFP are related to the free volume change in the EPDM/WHF composites. That could also be connected to the specific density of the sample wherever high density leads to a large value of µ_L_ and low values of HVL and MFP, similar to the data of Willner et al.^53^. The present data of γ-transmission agrees with the data deduced from PALS, where the EW30 has a smaller fraction of the free volume (relatively high density) compared with those of the other samples.

**Table 2:** The γ-ray attenuation parameters [the linear attenuation coefficient (μ_L_), the half value layer (HVL), and the mean free path (MFP)] at an energy of 59.5 keV of ^241^Am for the unirradiated samples.

Samples	µ (cm^-1^)	x_1/2_ (HVL) (cm)	MFP (cm)
SW30	0.01722 ± 0.00086	40.24 ± 2.01	58.07 ± 2.90
EW30	0.03511 ± 0.00176	19.74 ± 0.99	28.48 ± 1.42
HW30	0.01904 ± 0.00095	36.40 ± 1.82	52.52 ± 2.63

**Fig. 4:**
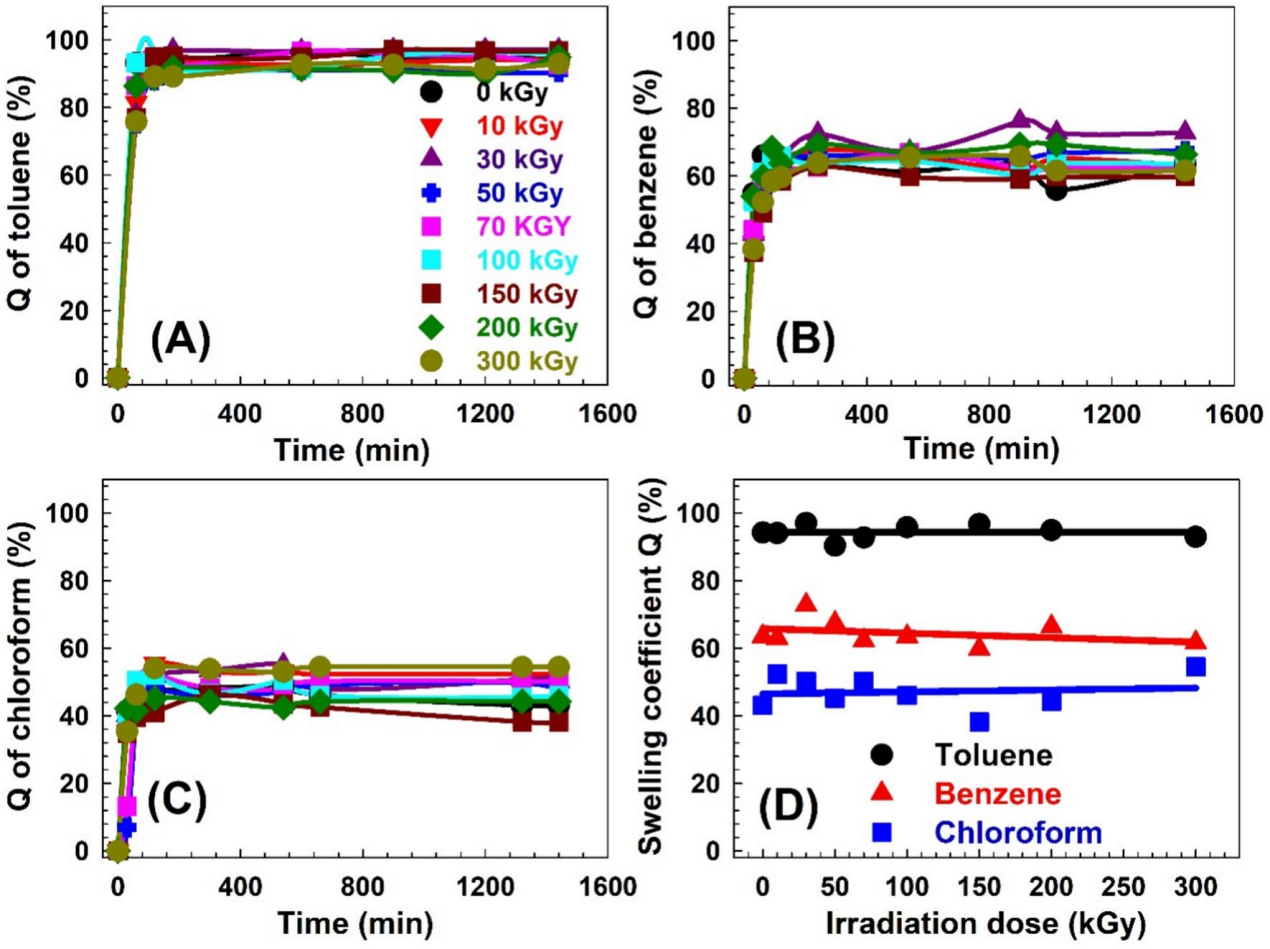
(**A**)–(**C**) The swelling coefficient Q for the SW30 samples as a function of time for the three solvents. (**D**) The swelling coefficient Q for the three solvents as a function of γ-irradiation doses for the SW30 samples.

**Fig. 5:**
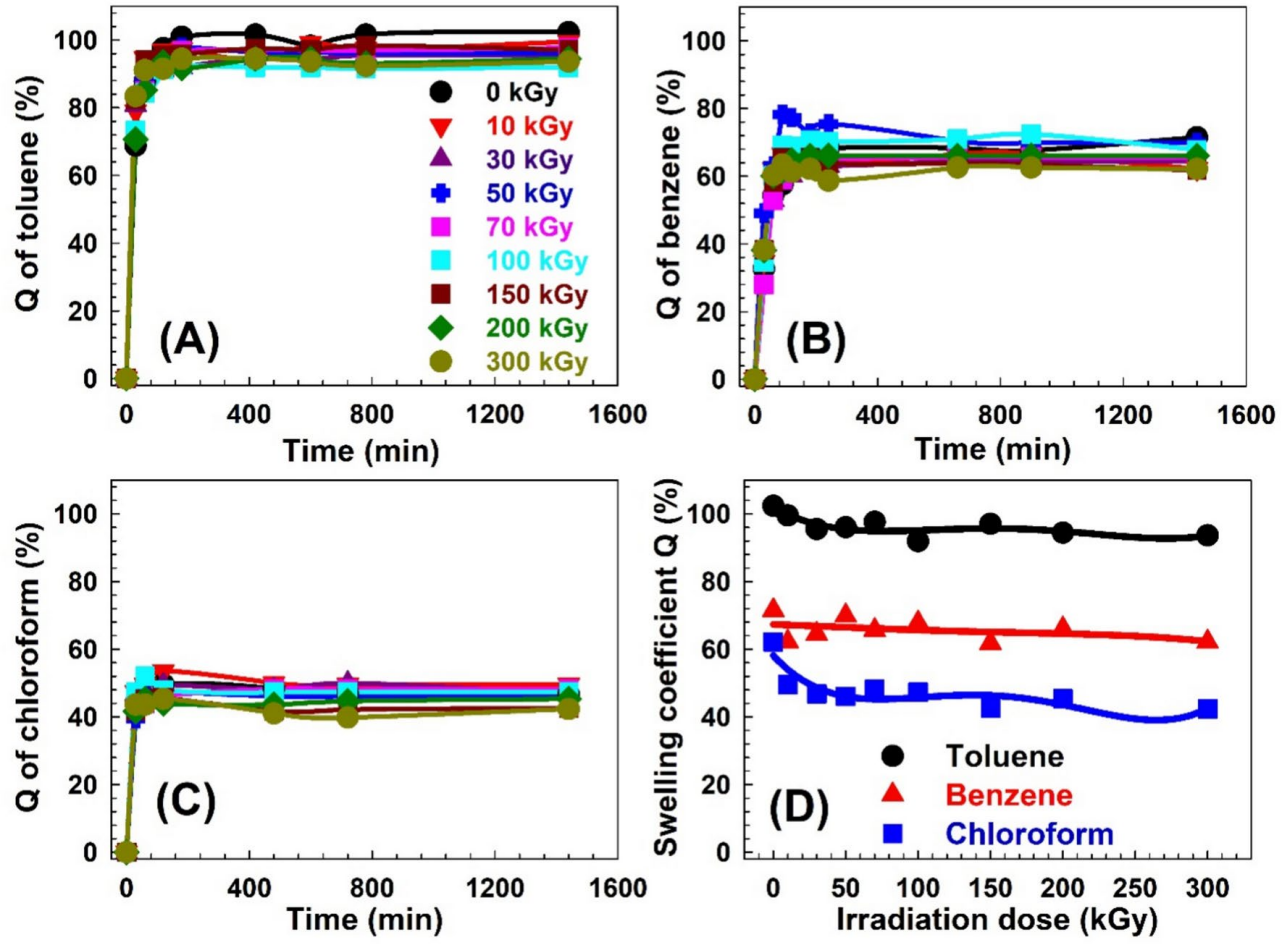
(**A**)–(**C**) The swelling coefficient Q for the EW30 samples as a function of time for the three solvents. (**D**) The swelling coefficient Q for the three solvents as a function of γ-irradiation doses for the EW30 samples.

**Fig. 6:**
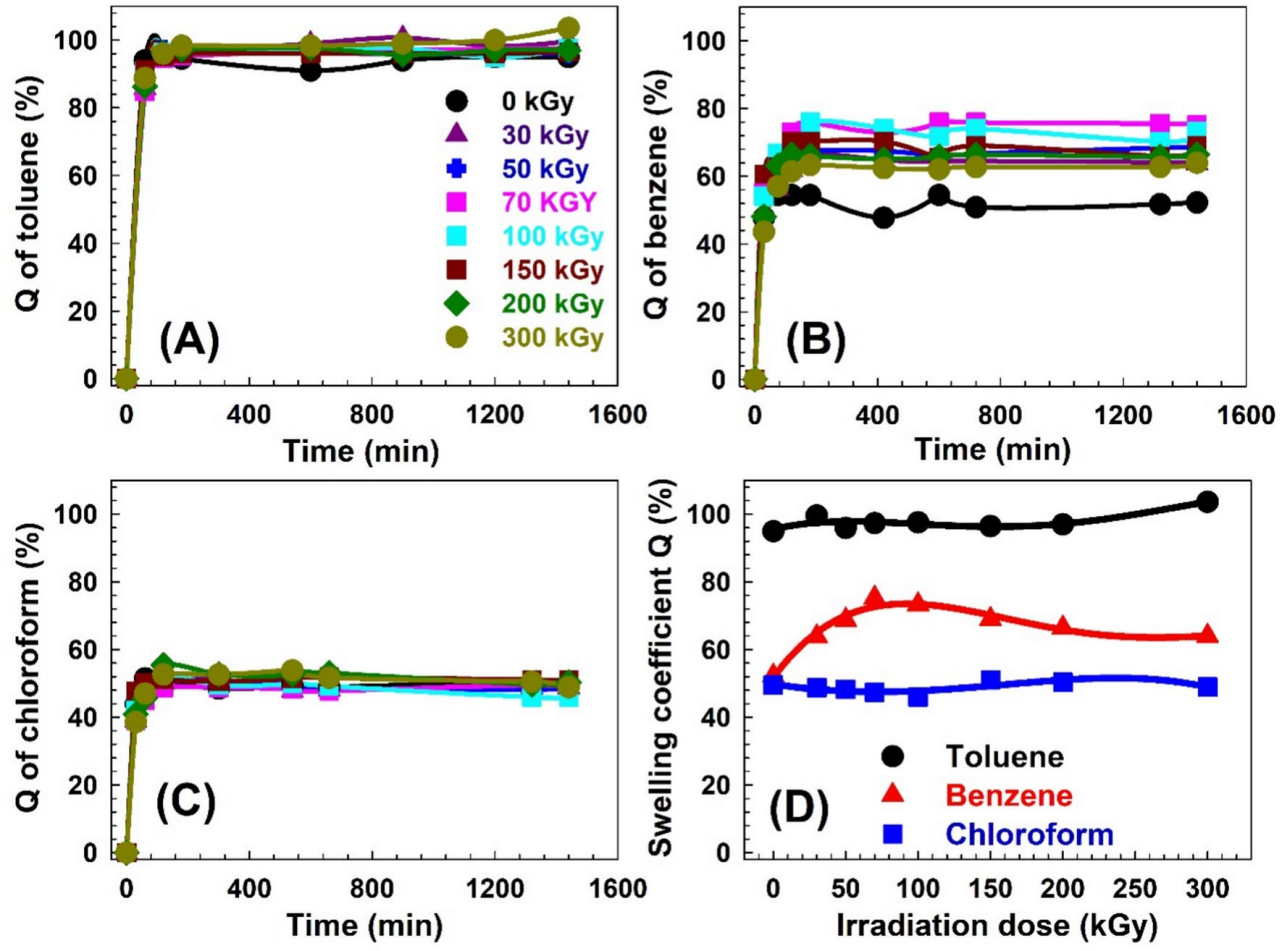
(**A**)–(**C**) The swelling coefficient Q for the HW30 samples as a function of time for the three solvents. (**D**) The swelling coefficient Q for the three solvents as a function of γ-irradiation doses for the HW30 samples.

The swelling behavior of solvents in polymers is a fundamental aspect of their physical and chemical properties, impacting their performance in various uses. Understanding and characterizing the swelling ratio of solvents in polymers is crucial for optimizing their design and predicting their behavior in different environments. The swelling ratio of solvent in a polymer is a measure of its ability to absorb and retain a solvent or liquid within its structure^54^ and it depends on several factors, including the polymer’s chemical composition, molecular weight, crosslinking density, and the nature of the solvent or liquid medium^55^. The swelling coefficient Q represents the relative change in weight of the polymer sample due to solvent absorption. It is typically expressed as a decimal or percentage. A positive value of Q indicates an increase in weight and volume, indicating swelling, while a negative value of Q suggests contraction or shrinkage of the polymer. Figures 4, 5 and 6 show the swelling coefficient Q for toluene, benzene, and chloroform for the SW30, EW30, and HW30, respectively, at different swelling times and γ-irradiation doses. The behaviors of the Q with the time are similar for all the samples at swelling solvents and at different irradiation doses. The Q value increases quickly with increasing the swelling time (30–60 min) and then levels off with increasing the time of swelling up to 1440 min. This pattern can be attributed to several underlying factors that influence the diffusion and absorption of the solvent’s molecules within the polymer matrix^56^. During the swelling time within 30–60 min, the solvent uptake in the polymer exhibits a steep rise. This rapid increase in Q can be attributed to the availability of numerous diffusion pathways and unoccupied binding sites within the polymer matrix. The polymer’s structure and composition at this stage allow for efficient penetration and diffusion of solvent molecules, leading to a significant increase in uptake within the initial period^57^. As time progresses beyond the initial 30–60 min, the solvent uptake reaches saturation and remains Q relatively constant until 1440 min. This constant solvent uptake signifies the establishment of equilibrium between the concentration of solvent in the surrounding environment and the concentration within the sample^58^. At this point, the rate of solvent diffusion into the polymer becomes balanced with the rate of solvent desorption or evaporation from the polymer surface. The plateau phase suggests that the polymer has reached its maximum capacity for solvent absorption under the given conditions. The sequence of Q is Q (toluene) > Q (benzene) > Q (chloroform) for all the samples and also for all γ-irradiation dose ranges. This behavior of the Q can be attributed to several factors, including the nature of the solvents, such as molecular size and polarity, and the specific characteristics of the composite material made from a combination of natural fibers (WHF) and synthetic polymers (EPDM). For the molecular size, benzene is a cyclic hydrocarbon consisting of six carbon atoms and six hydrogen atoms. It has a symmetrical structure, and its molecular size is smaller compared to toluene. In addition, toluene is a benzene ring with a methyl group attached, where the presence of the methyl group increases the molecular size to 0.177 nm^3^^59^ compared to benzene 0.119 nm^3^^59^. Moreover, chloroform consists of one carbon atom, one hydrogen atom, and three chlorine atoms, where the molecular size of the chloroform is 0.102^60^ or 0.104 nm^3^^61^. So, the sequence of the solvent molecular size is toluene > benzene > chloroform. Small molecular size could be absorbed more than large molecular size; however, this behavior does not work here. The present samples are swelling more with bigger molecular size (toluene) compared with that for smaller molecular size (benzene or chloroform). Also, the average free volume size of the amorphous regions deduced from PAL techniques showed that it is in the range from 0.092 to 0.157 nm^3^. Because the samples are swollen, the free volume would be expected to be larger, and thus, chloroform would be easier to transport in the free volume of the samples, but transport of toluene would be more difficult. Therefore, the relationship between the molecular size of the solvents and the free volume of the samples resulted in slow diffusion, in contrast to the diffusivity of toluene.

Let’s look at each solvent separately: Toluene is a methyl group-attached benzene ring. As the methyl group donates electrons, increasing the ring’s electron density, it is more reactive than a typical benzene ring. Electron-donating groups increase the reactivity of the main group through a positive inductive effect. Electron-withdrawing groups do the opposite. In addition, toluene is a relatively nonpolar solvent with a polarity of 2.4, and it exhibits favorable interactions with nonpolar materials^62^. In the case of the composite material composed of natural fibers and synthetic polymers, the nonpolar nature of toluene with a lower polarity number allows for stronger interactions with the nonpolar components of the composite. These hydrophobic components can effectively interact with toluene through van der Waals forces or other nonpolar interactions, leading to increased absorption. Additionally, the synthetic polymer EPDM, being a hydrophobic material, can also contribute to the enhanced absorption of toluene (Fig. 4). Then benzene is another nonpolar solvent with similar characteristics to toluene, and its polarity is 3^63^. However, compared to toluene, benzene may exhibit weaker interactions with the composite material. This weaker interaction could be due to the specific structural features of benzene or the composite’s composition. While the nonpolar components of the composite material can still interact with benzene to some extent, the strength of these interactions might be relatively weaker than that for toluene (Fig. 5). Consequently, the composite material absorbs less benzene compared to toluene. At least chloroform is a polar solvent, and its interactions with the composite material are primarily governed by polar forces such as dipole–dipole interactions or hydrogen bonding^64^. The composite material, which consists of natural fibers and synthetic polymers, may have limited polar components or functional groups available for strong interactions with chloroform^65^. Natural fibers, such as WHF, may contain polar groups. However, these polar groups may not be as abundant or accessible in the composite material, leading to weaker interactions with chloroform (Fig. 6). Additionally, the synthetic polymer EPDM, being hydrophobic in nature, may have limited compatibility with the polar chloroform molecules, further reducing the absorption capacity^66^.

Figures 4D, 5D and 6D present the swelling coefficient Q after 1440 min (saturation point) for all the solvents as a function of γ-irradiation doses for SW30, EW30, and HW30, respectively. It is clear from Fig. 4D that the behavior of Q is almost constant without significant change with irradiation dose for SW30 samples, where it is 95, 65, and 46% for toluene, benzene, and chloroform, respectively. The unchanged behavior can be connected to the degradation effects that take place in the samples because of γ-irradiation doses and the polymer composite swollen with the solvents in the saturation effects. For the EW30 samples (Fig. 5D), the trends of the Q with γ-irradiation doses are like the trends of the free volume size (Fig. 2). The Q values decrease with the irradiation dose up to 50 kGy and then they level off. It is well known that there is a linear correlation between the sorption or swelling and the free volume hole in polymer like the data of Ito et al.^67^. Figure 6D shows the Q values as a function of the irradiation dose for the HW30 samples. The behaviors for toluene and chloroform are similar to those for the SW30 samples, where the Q increases (75%) with increasing the irradiation dose up to 100 kGy and then decreases smoothly with the irradiation dose (64%). The interpretation of this trend cannot be explained in terms of the free volume model and needs more investigation.

Studying the alternating current (*AC*) conductivity conveys the conduction mechanism in a material by applying *AC* signal to the material under investigation and measuring its response with different frequencies. The *AC* conductivity as a function of frequency can be clarified in terms of Jonscher’s universal power law^26,68,69^:

8 $${\sigma }_{AC}=B{\omega }^{s}$$

**Fig. 7:**
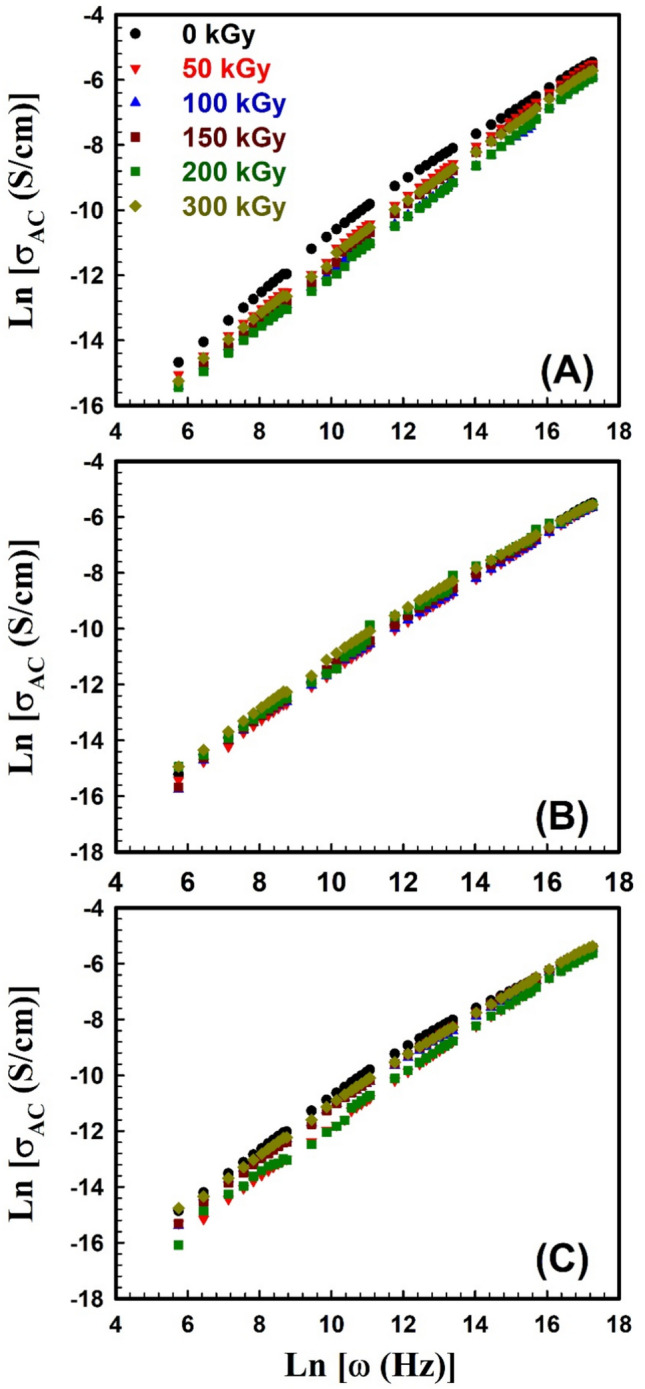
Frequency dependence of *AC* electrical conductivity σ_AC_ for (**A**) SW30, (**B**) EW30, and (**C**) HW30 samples at different γ-irradiation doses.

**Fig. 8:**
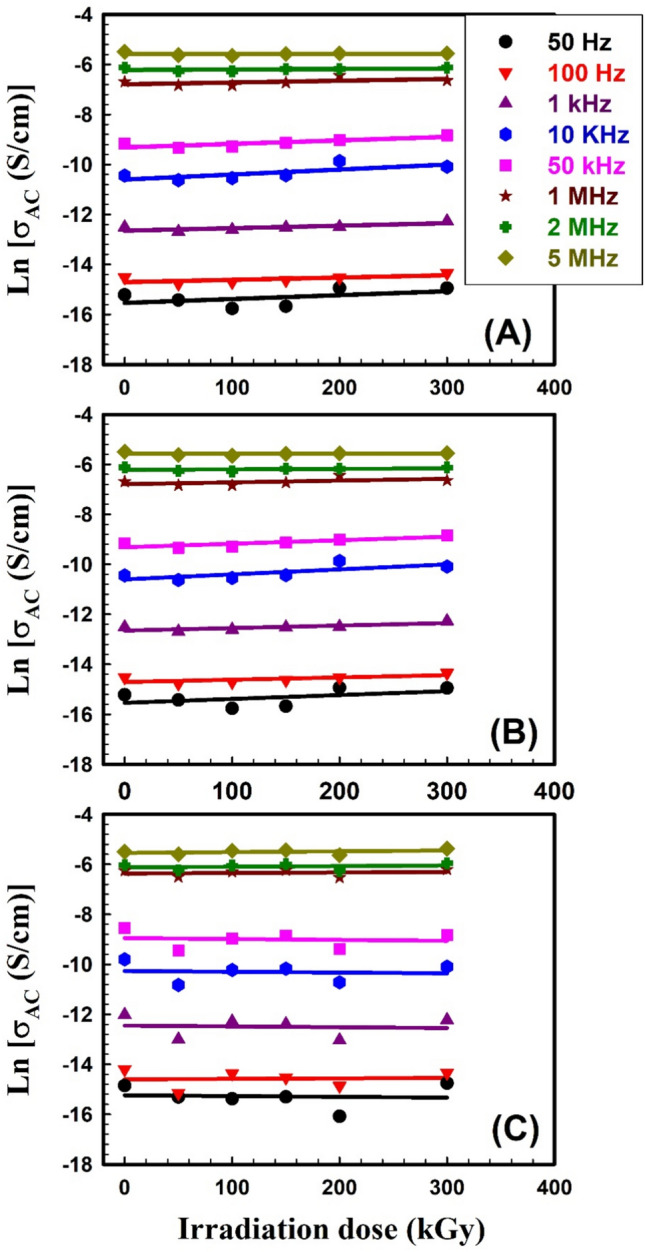
The *AC* electrical conductivity σ_AC_ as a function of the γ-irradiation dose for (**A**) SW30, (**B**) EW30, and (**C**) HW30 samples at different frequencies.

The constant *B* is depending on temperature, *ω* is the angular frequency (2π*f*), and *s* is the angular frequency exponent. The *AC* electrical conductivity characterization of EPDM/WHF composites was measured in the frequency range from 50Hz to 5MHz and at different γ-irradiation doses. Figure 7A–C depicts the relation between ln *σ*_*AC*_ against ln *ω* for SW30, EW30, and HW30 composites, respectively, with different γ-irradiation doses at room temperature. It was seen that the *AC* conductivity increases with increasing frequency, for all irradiation doses. This behavior may be due to the enhancement of conductive pathways for charge carriers and ions^70^. This is consistent with the behavior of free volume. On one hand, the relation between *AC* conductivity versus γ-irradiation doses is depicted in Fig. 8A–C for SW30, EW30, and HW30 composites, respectively. It’s clear from the figure that the *AC* conductivity slightly decreases with increasing irradiation doses up to 300 kGy. The reduction in *AC* conductivity is described by limiting charge carrier mobility through the enhancement of crosslinking density of the polymer chains by irradiation^70^, and due to the recombination of free ions because of dimer formation^71^. In addition, the differences in the intrinsic conduction properties of the media can affect the conductivity of the prepared composites. This behavior is compatible with that for some EPDM/flax fiber composites after exposed to different doses of electron beam irradiation^72^. It is important to elucidate the value of the angular frequency exponent *s* which explores the conduction relaxation mechanism in non-ordered polymers. The *s* values are calculated from the slope of the obtained straight lines in Fig. 7. The obtained values of *s* for all composites under investigation are tabulated in Table 3. The behavior of *s* with different doses looks similar to that of the relation between free volumes versus different doses. The calculated *s* values are found to be less than unity and decrease with increasing irradiation doses. This supposes that the conduction is ruled by a hopping mechanism in these composites (correlated barrier hoping model, CBH model). The CBH model describes the hopping of charge carriers between two sites over a barrier separating them. According to this model, the barrier height *W*_*m*_ (the maximum barrier height at infinite separation), can be calculated at room temperature (*T* = 300 K) for all composites under investigation using the first approximated equation as;

9 $$W_{m} = \frac{{6k_{B} T}}{1 - s}$$

where *k*_*B*_ is the Boltzmann constant. The estimated values of *W*_*m*_ of all composites at different irradiation doses are given, also, in Table 3.

**Table 3:** Values of frequency exponent (*s*) and the barrier height (*W*_M_) for all composites under investigation at different γ-irradiation doses.

Samples (kGy)	SW30		EW30		HW30	
	*s*	*W*_*M*_ (eV)	*s*	*W*_*M*_ (eV)	*s*	*W*_*M*_ (eV)
0	0.86	1.10	0.83	0.91	0.80	0.78
50	0.85	1.04	0.84	0.92	0.88	1.22
100	0.82	0.86	0.83	0.91	0.82	0.86
150	0.85	1.04	0.84	0.92	0.84	0.92
200	0.83	0.91	0.83	0.91	0.87	1.13
300	0.82	0.86	0.81	0.82	0.82	0.86

**Fig. 9:**
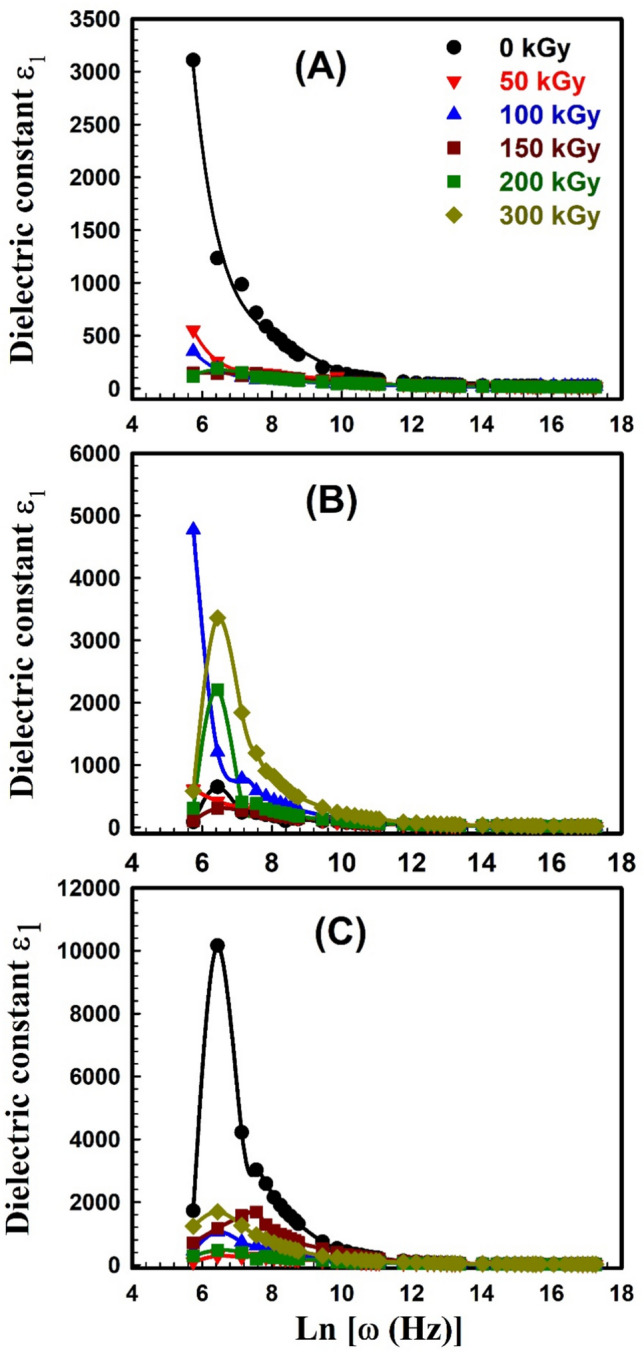
Variation of dielectric constant ε_1_ with frequency for (**A**) SW30, (**B**) EW30, and (**C**) HW30 samples at different γ-irradiation doses.

**Fig. 10:**
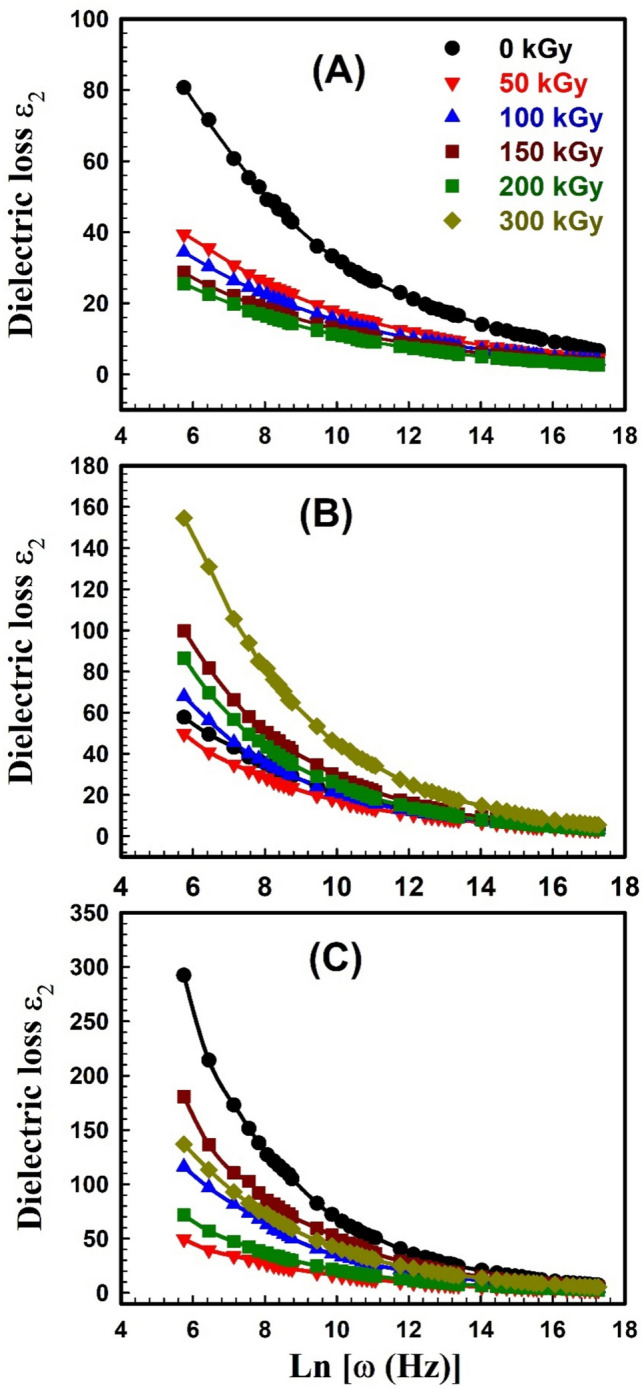
Variation of dielectric loss ε_2_ with frequency for (**A**) SW30, (**B**) EW30, and (**C**) HW30 samples at different γ-irradiation doses.

**Fig. 11:**
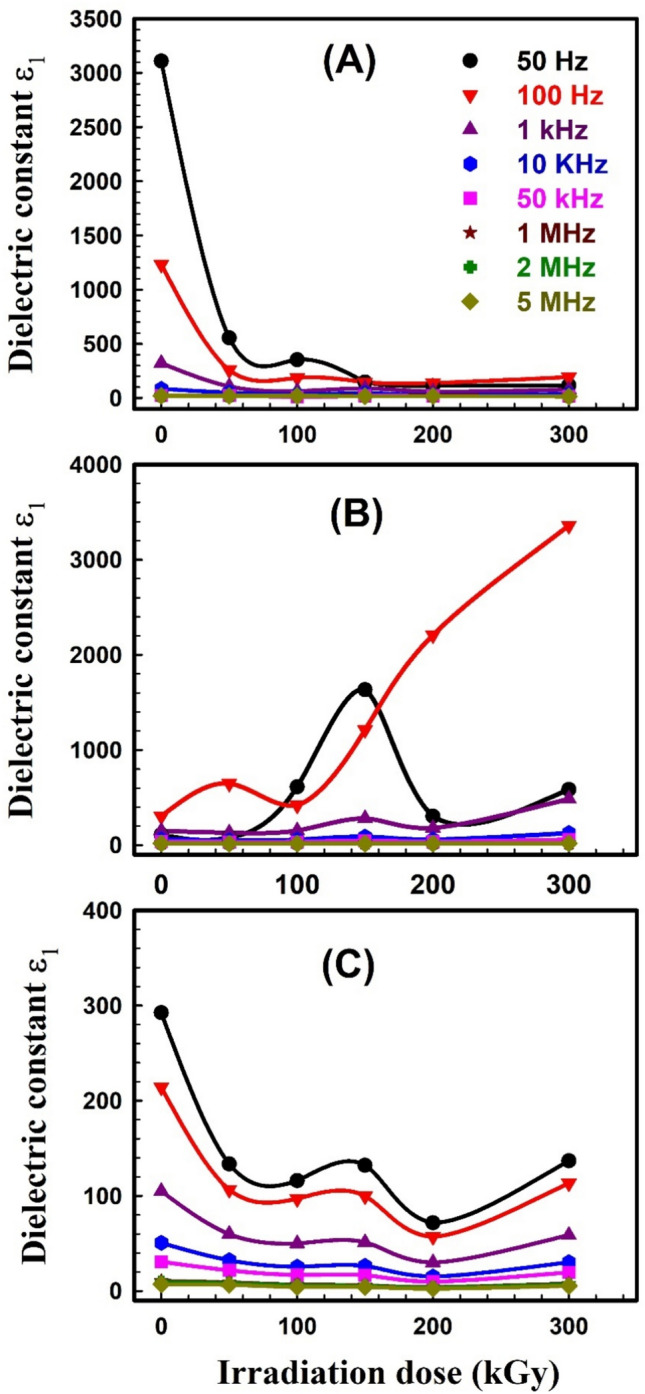
Dielectric constant ε_1_ as a function of γ-irradiation doses for (**A**) SW30, (**B**) EW30, and (**C**) HW30 samples at different frequencies.

**Fig. 12:**
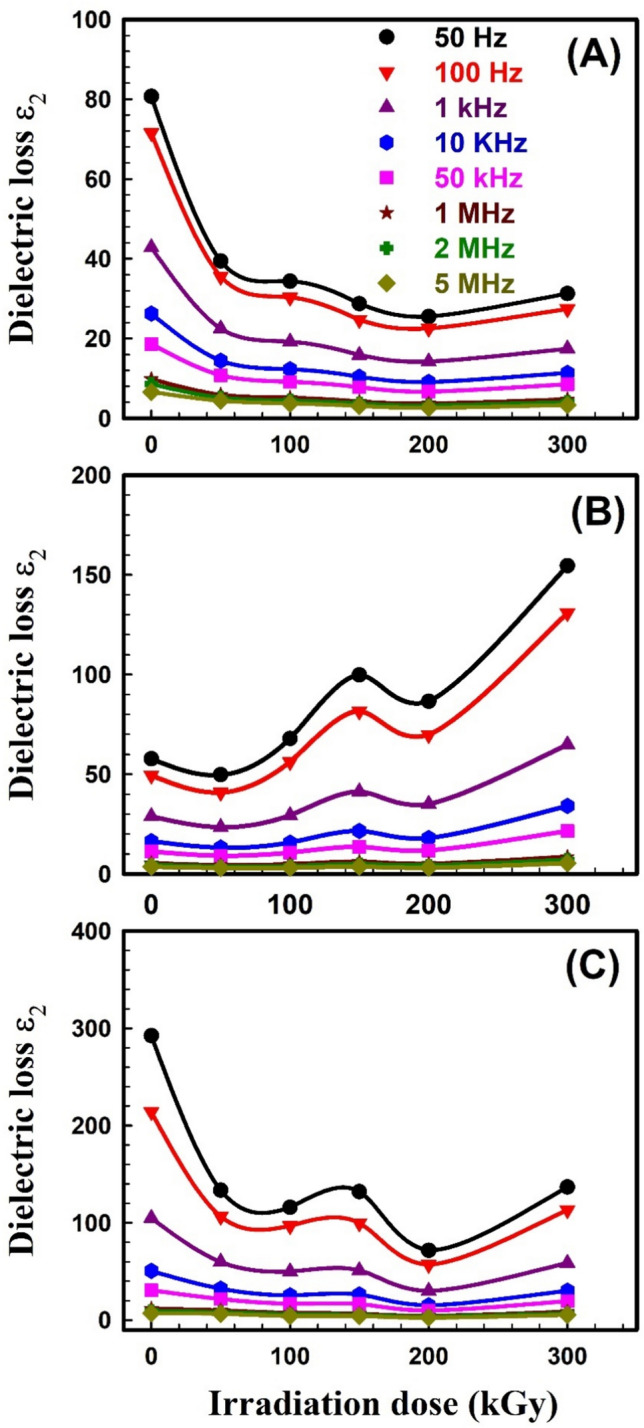
Dielectric loss ε_2_ as a function of γ-irradiation doses for (**A**) SW30, (**B**) EW30, and (**C**) HW30 samples at different frequencies.

The dielectric behavior of any polymer is described by two parameters; 1) the dielectric constant *ε*_*1*_ which is related to the capacitive nature of the medium and reveals the relation between the polarization of a material and the applied electric field, and 2) the dielectric loss *ε*_2_ which describes the dissipation energy that characterizes the losses in a material. Dielectric spectroscopy was done to explore the impact of the electric field on unirradiated and irradiated SW30, EW30, and HW30 composites. The complex dielectric ε* with its two parameters *ε*_1_ and *ε*_2_ can be expressed as:

10 $$\varepsilon^{ * } \left( \omega \right) = \varepsilon_{1} \left( \omega \right) + i\varepsilon_{2} \left( \omega \right)$$

11 $${\varepsilon }_{1}=\frac{Ct}{{\varepsilon }_{o}A}$$

12 $$\varepsilon_{2} = \varepsilon_{1} \tan \delta$$

where *t* is the thickness, *C* is the capacitance, *ε*_o_ is the vacuum permittivity, and δ (loss factor) equals 90-θ and θ is the phase angle. The real part *ε*_1_ of dielectric permittivity for unirradiated and irradiated SW30, EW30, and HW30 composites at different doses, respectively, as a function of frequency; is depicted in Fig. 9A–C. The high values of *ε*_1_ are associated with the influence of the contribution of multiple types of polarizations at low frequency. The relation seems to be frequency-independent at high frequencies because of interfacial polarization since the dipoles can no longer catch the field. This is the main reason for a constant plateau that has appeared at high frequencies in all samples. The figure also concluded that the* ε*_1_ value decreases with γ-irradiation doses. Figure 10A–C illustrates the relation between *ε*_*2*_ versus frequency for unirradiated and irradiated SW30, EW30, and HW30 composites at different doses, respectively. The values of *ε*_2_ decrease as frequency increases, in all samples, because of ion transfers in the material, which is considered to be the main reason for this behavior at low frequencies. At high frequency, the independent relation between *ε*_2_ and ln*ω* relation is due to the reduction of dielectric losses in only ion vibration loss. In addition, the *ε*_2_ value decreases with γ-irradiation doses. The decrease in both the* ε*_1_ values for SW30 composite with increasing γ-radiation doses, as illustrated in Fig. 11A, can be attributed to the appearance of some imperfections through crosslinking in the composite under investigation. Accordingly, the defects repress the carrier’s movement, hence the polarization effect. For both EW30 and HW30 composites, the maximum value of dielectric constant is shown at a dose of 150 kGy, and then it decreases at 200 kGy, followed by another increase at 300 kGy, as illustrated in Fig. 11B and C. The inset figure in Fig. 11A describes the same relation at frequencies of 50 and 100 Hz. The same similar behavior is observed for the dielectric loss-radiation dependencies as depicted in Fig. 12A–C. This behavior is attributed to the multi-relaxation processes in the composites since the dielectric relaxation of composites is largely reliant on the polymer-husk interaction. In these polymer composites, the interfaces between different phases can contribute to interfacial polarization, where charges accumulate at the interface, enhancing the dielectric constant. Also, the dielectric loss can be understood as energy dissipated due to molecular friction during the reorientation process of the desired dipoles.

**Fig. 13:**
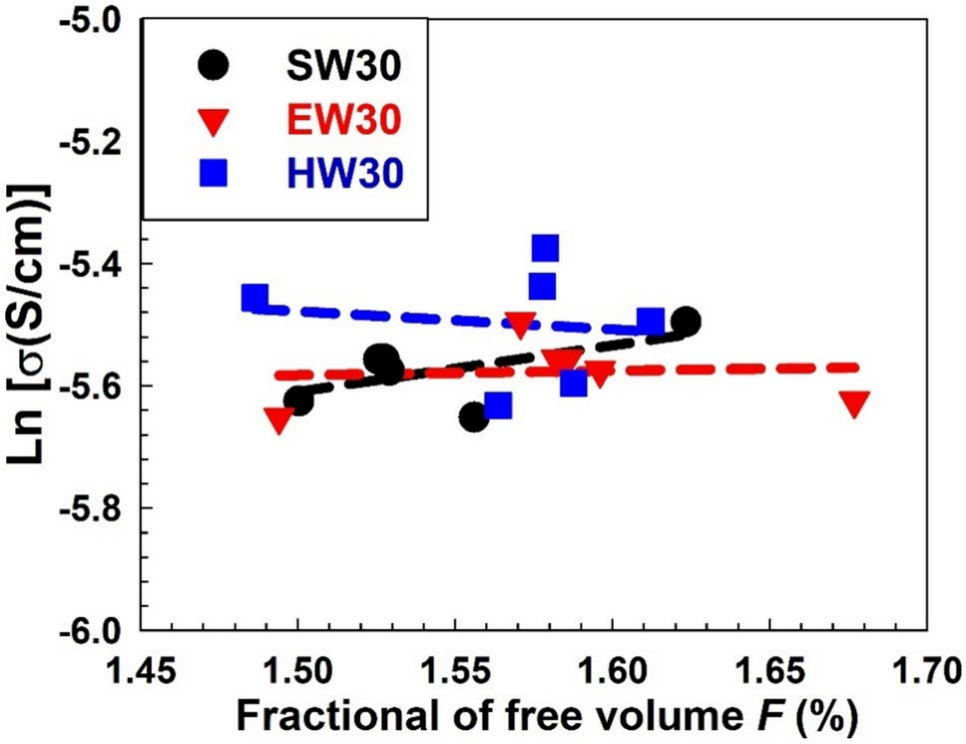
The correlation between the fractional of free volume *F* and the *AC* electrical conductivity σ_AC_ for the SW30, EW30, and HW30 composites. The broken lines are drawn just for clarification.

Using the *o*-Ps intensity *I*_3_ and free volume size* V*, the fractional free volume *F* is estimated according to the following equation:

13 $$F = C^{\prime } \;V\;I_{3} ,$$

where $C^{\prime}$ is empirically determined to be 1.8 nm^−3^ from the specific volume data^73^. It is interesting to study the correlation between the *AC* electrical conductivity σ_*AC*_ and the nanostructure of the composites deduced from PAL data, such as the fractional of free volume *F*. Figure 13 shows the correlation between the AC electrical conductivity and the fractional of free volume *F* for the SW30, EW30, and HW30 samples. There are linear correlations between them, indicating the ionic conservativity of the present composites is controlled by the free volume size. In addition, the slope of the correlation for each composite group is not the same, supporting that the free volume size affects not only the conduction mechanism of the conductivity but also the length of the WHF loaded in EPDM polymers.

## Conclusion

The results of the present study offer valuable insights into the effects of WHF length and γ-irradiation dosages on the free volume structure and properties of EPDM/WHF composites. Ionizing radiation has a significant impact on the composites under study, leading to changes in the free volume structure, chain scission, fragmentation of molecules, and crosslinking. Increasing the free volume hole size at low γ-irradiation doses are connected to the degradation effect on the SW30 samples. While decreasing the *o*-Ps intensity on the SW30 samples with γ-irradiation dose is connected to both effects: formation of free radicals or increase on the degree of crystallinity by γ-irradiation. For the EW30 and HW30, the shrinkage of free volume in the composites is primarily due to crosslinking induced by γ-irradiation. The formation of voids or defects within the composites due to irradiation leads to an increase in the free volume contents. The μ_L_ value for the EW30 is lower compared with those for the SW30 and HW30; on the other hand, the EW30 has a smaller fraction of the free volume compared with those of the other samples. The solvents’ entire swelling coefficients rise with extended swelling times before levelling out; this is dependent on the solvent’s structure and crosslinking density. The *AC* conductivity increases with increasing frequency due to the enhancement of conductive pathways for charge carriers and ions. The *AC* conductivity showed a frequency-dependent increase, indicating enhanced charge carrier mobility. The conduction mechanisms are found to be quantum mechanical tunnelling and correlated barrier hopping, depending on the composite and γ-irradiation dose. The *AC* conductivity slightly decreases with increasing γ-irradiation doses up to 300 kGy. Those are due to limiting charge carrier mobility through the enhancement of crosslinking density of the polymer chains by irradiation, and due to the recombination of free ions as a result of dimer formation. There is a good correlation between the conductivity results and the fractional of free volume deduced from the PAL technique, indicating that the conductivity is supported by the free volume structure in the composites.

## Data Availability

The datasets used and/or analyzed during the current study are available from the corresponding author on reasonable request.
